# Research progress of ferroptosis in acute kidney injury

**DOI:** 10.3389/fcell.2025.1614156

**Published:** 2025-06-25

**Authors:** Lin Zhang, Feng Luo, Nan Yuan, Jiaming Yin, Bing Shen, Yalin Chai, Lijie Sun, Xuan Wang, Le Yin, Congjuan Luo

**Affiliations:** ^1^ Department of Nephrology, The Affiliated Hospital of Qingdao University, Qingdao, Shandong, China; ^2^ Department of Cardiology, The Affiliated Hospital of Qingdao University, Qingdao, Shandong, China

**Keywords:** acute kidney injury, ferroptosis, lipid peroxidation, mechanisms, therapeutic targets

## Abstract

Acute kidney injury (AKI) is a life-threatening condition characterized by a rapid decline in kidney function caused by various underlying factors. Despite advancements in medical science, effective treatments for AKI remain limited, highlighting the necessity for novel therapeutic strategies. Ferroptosis, an iron-dependent regulated cell death characterized by lipid peroxidation, has been recently linked to AKI development. Studies indicate that ferroptosis plays a role in multiple AKI types, such as those caused by ischemia-reperfusion, sepsis, nephrotoxic agents, and rhabdomyolysis. In these conditions, ferroptosis markers are elevated in renal tubular epithelial cells, and inhibiting ferroptosis has been shown to reduce kidney injury. However, the precise regulatory mechanisms of ferroptosis in AKI remain unclear. This review summarizes current understanding of ferroptosis, including its definition, molecular regulation, involvement in various AKI types, and potential therapeutic targets. By elucidating these aspects, we hope to provide a foundation for future research and the development of effective interventions for AKI.

## 1 Introduction

AKI is a clinical syndrome marked by a sudden decrease in kidney function within hours to weeks, resulting in damage to renal tubular epithelial cells. Epidemiological studies show that AKI is a prevalent and severe complication in hospitalized patients, with an incidence of approximately 10%–15%, and rising to over 50% in intensive care unit (ICU) patients (Al-Jaghbeer et al., 2018). The development of AKI involves multifactorial etiologies, including ischemia-reperfusion injury, systemic infections leading to sepsis, exposure to nephrotoxic agents, and rhabdomyolysis. Each of these factors contributes to renal tissue damage through distinct yet interrelated mechanisms, such as oxidative stress, inflammation, and direct cytotoxicity, thereby complicating the clinical management of AKI (Basile et al., 2012; Ow et al., 2021). Currently, clinical management of AKI primarily relies on renal replacement therapies like hemodialysis, with limited alternative treatments. Consequently, there is a critical need to elucidate the molecular and cellular mechanisms underpinning AKI to identify novel therapeutic targets and develop effective preventive strategies.

Ferroptosis is a distinct form of regulated cell death characterized by iron-dependent accumulation of lipid peroxides, leading to oxidative damage of cellular membranes. Unlike apoptosis or necrosis, ferroptosis involves specific metabolic pathways, including iron metabolism and lipid peroxidation, and is regulated by key enzymes such as glutathione peroxidase 4 (GPX4). Research has demonstrated that during AKI, there is a significant upregulation of ferroptotic activity within renal tubular epithelial cells. This increase in ferroptosis correlates with the severity of tissue damage, suggesting a contributory role in the pathophysiology of AKI. Experimental studies have shown that pharmacological agents, including iron chelators and specific ferroptosis inhibitors, can mitigate renal damage in various AKI models. These findings suggest that therapeutic strategies aimed at modulating ferroptosis pathways may offer protective benefits against AKI-induced renal injury (Martin-Sanchez et al., 2017). Therefore, exploring the molecular mechanisms of ferroptosis and its intervention strategies holds significant clinical importance. This article reviews the mechanisms of ferroptosis in AKI, the latest research developments, and its potential as a therapeutic target. The aim is to provide new insights into future research directions for improving the treatment of AKI.

## 2 Definition and regulatory mechanisms of ferroptosis

Ferroptosis is an iron-dependent form of programmed cell death characterized by the accumulation of lipid peroxides. Ferroptosis differs markedly from other forms of programmed cell death, such as apoptosis, necroptosis, and autophagy in mechanisms, morphology, and genetics (Table 1). Biochemically, ferroptosis is marked by reduced intracellular glutathione (GSH) levels, suppressed GPX4 activity, and dysregulated iron homeostasis (Zheng and Conrad, 2020). Morphologically, ferroptosis is characterized by a reduction in mitochondrial number and volume, increased membrane density, and loss of mitochondrial cristae, while cell membrane integrity and nuclear morphology remain intact (Wang et al., 2020). Genetically, ferroptosis is regulated by multiple genes and involves aberrant expression linked to the iron homeostasis system, amino acid metabolism, and oxidative stress induced by lipid radicals. The mechanisms of ferroptosis are complex and warrant further investigation (Yan et al., 2021).

**TABLE 1:** The main comparison of different types of cell death.

Cell death type	Biochemical features	Morphological features	Key genes
Ferroptosis	Iron-dependent, non-apoptotic cell death characterized by glutathione depletion, GPX4 inactivation, ROS accumulation, and lipid peroxidation	Mitochondrial shrinkage, increased membrane density, cristae loss, and plasma membrane rupture with an intact nucleus	*GPX4, TFR1, ACSL4, SLC7A11, NRF2*
Apoptosis	Caspase-mediated cell death involving DNA fragmentation and mitochondrial membrane potential loss	Cell shrinkage, membrane blebbing, chromatin condensation, nuclear fragmentation, and apoptotic body formation	*BCL-2, CASPASES, TP53, FAS, FASL*
Necroptosis	RIPK1/RIPK3-mediated and ROS-dependent cell death accompanied by ATP depletion and DAMP release	Plasma membrane rupture, cytoplasmic and organelle swelling, moderate chromatin condensation, and intracellular content release	*RIPK1, RIPK3, CASPASE-8, BECN1, MLKL*
Pyroptosis	Caspase-1-dependent, gasdermin-mediated lytic cell death characterized by inflammasome assembly and pro-inflammatory cytokine release	Cell swelling, plasma membrane rupture, chromatin condensation, and cytokine release	*GSDMD, IL1B, CASPASE-1, NLRP3*
Autophagy	Lysosome-dependent and ATP-driven degradation of cytoplasmic components	Formation of double-membrane autophagosomes and autolysosomes via macroautophagy, microautophagy, and chaperone-mediated autophagy	*ATG5, ATG7, BECN1, LC3*

**FIGURE 1:**
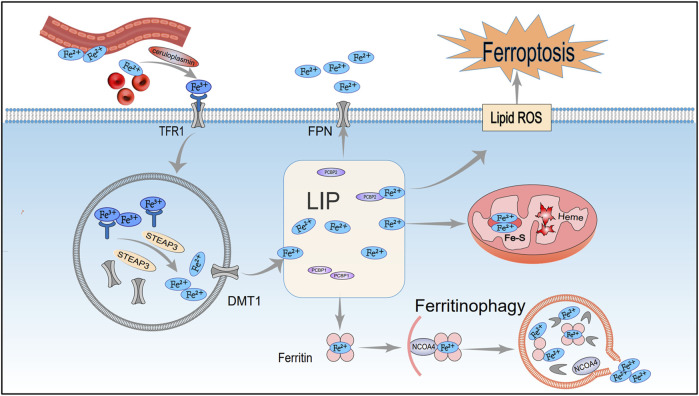
Mechanisms of iron metabolism regulating ferroptosis. After entering the cell via TFR1, extracellular iron ions undergo complex reactions in organelles such as the nucleus, mitochondria, and lysosomes, facilitating the maintenance of iron homeostasis. However, under pathological conditions, disruptions in iron metabolism can trigger lipid peroxidation and ferroptosis. TFR1, Transferrin receptor 1; STEAP3, Endosomal iron reductase; DMT1, Divalent metal transporter 1; LIP, Labile iron pool; Fe-S, Iron-sulfur cluster; FPN1, Ferroportin 1; NCOA4, Nuclear receptor co-activator 4.

**FIGURE 2:**
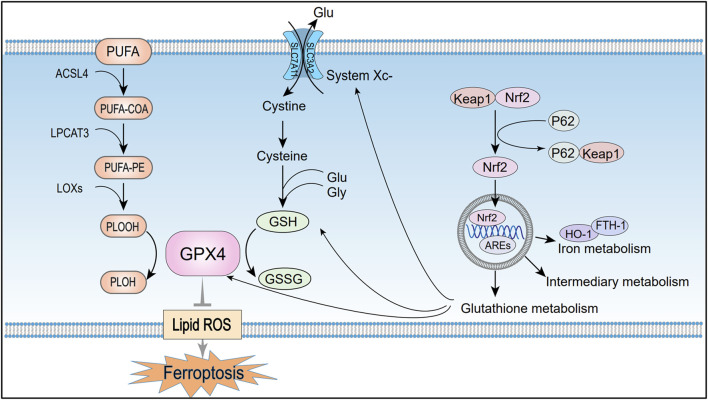
Mechanisms of ferroptosis regulation by lipid metabolism, amino acid metabolism, and the p62-Keap1-Nrf2 pathway. Under the combined catalytic action of ACSL4, LPCAT3, and LOX, PUFAs generate peroxides that trigger free radical chain reactions and accelerate ferroptosis. The System Xc−-GSH-GPX4 metabolic axis, which is formed during amino acid metabolism, is a crucial pathway for regulating ferroptosis. Additionally, Nrf2 maintains cellular redox balance and protects against ferroptosis by activating gene expression through AREs. PUFA, Polyunsaturated fatty acids; PUFA-COA, Acyl-CoA derivatives; PUFA-PE, Phosphatidylethanolamine; PLOOH, Lipid peroxides; PLOH, Corresponding alcohols of lipid peroxides; ACSL4, Long-chain acyl-CoA synthetase member 4; LPCAT3, Lysolipid acyltransferase 3; LOXs, Lipoxygenases; GPX4, Glutathione peroxidase 4; System Xc−, Cystine/glutamate antiporter; SLC7A11, Solute carrier family seven member 11; SLC3A2, Solute carrier family 3 member; Glu, Glutamate; GSH, Reduced glutathione; GSSG, Oxidized glutathione; Lipid ROS, Lipid reactive oxygen species; Nrf2, Nuclear factor erythroid 2-related factor; KEAP1, Kelch-like ECH-associated protein 1; P62, Sequestosome 1 (SQSTM1); AREs, Antioxidant response elements; HO-1, Heme oxygenase-1; FTH1, Ferritin heavy chain 1.

Recent studies have shown that ferroptosis is primarily driven by dysregulated iron metabolism, lipid peroxidation, amino acid metabolic imbalances, and abnormalities in the p62-Keap1-Nrf2 pathway. These mechanisms are shown in Figures 1, 2. A systematic investigation of these interconnected mechanisms may uncover novel targets for mitigating ferroptosis-related pathologies.

### 2.1 Iron metabolism

Iron is an essential element required for normal biological functions, and any abnormal distribution or concentration of iron ions can disrupt normal physiological functions. Under physiological conditions, Fe^2+^ from intestinal absorption or red blood cell turnover is oxidized to Fe^3+^ by ceruloplasmin (van Swelm et al., 2020). Circulating Fe^3+^ initially binds transferrin (TF) to form a TF–Fe^3+^ complex. It then binds transferrin receptor 1 (TFR1) to create TF–Fe^3+^–TFR1, which is internalized by receptor-mediated endocytosis (Philpott et al., 2020; Roemhild et al., 2021). Inside the endosome, Fe^3+^ is reduced to Fe^2+^ by STEAP3 and then passes into the cytosol via DMT1 channels, where it is stored temporarily in the labile iron pool (LIP) (Chen et al., 2020). Most of the iron in the LIP is transported into mitochondria for heme synthesis or iron–sulfur cluster assembly, while the remainder is sequestered by ferritin into a stable complex (Muckenthaler et al., 2017).

Ferritin is a multimeric iron-storage protein assembled from heavy (FTH1) and light (FTL) subunits. FTH1, as the core component of ferritin, can oxidize Fe^2+^ to Fe^3+^, which reduces free iron and preserves intracellular iron balance (Li and Li, 2020). During this process, nuclear receptor coactivator 4 (NCOA4) acts as an adaptor protein, regulating ferritin autophagy to maintain iron homeostasis. NCOA4 targets ferritin to lysosomes for degradation, releasing Fe^2+^. Studies show that reduced NCOA4 expression decreases labile iron and ROS accumulation, inhibiting ferroptosis, whereas NCOA4 overexpression accelerates ferritin degradation and promotes ferroptosis (Hou et al., 2016).

Moreover, pathological conditions characterized by low FTH1 expression alongside high TFR1 and NCOA4 levels disrupt iron metabolism, resulting in intracellular free iron accumulation. This excess free iron then drives Fenton reactions to produce ROS and hydroxyl radicals (OH^−^), which attack membrane polyunsaturated fatty acids (PUFAs), causing oxidative stress, lipid peroxidation, and ferroptosis (Chen et al., 2021) (Figure 1).

### 2.2 Lipid metabolism

Lipid peroxidation is a hallmark of ferroptosis, referring to the oxidation of lipids into peroxides via enzymatic or non-enzymatic pathways. Notably, membrane phospholipids are enriched in PUFAs, whose unstable carbon–carbon double bonds render them more prone to peroxidation than saturated or monounsaturated fatty acids (Dixon et al., 2012). Therefore, the abundance and distribution of PUFAs influence the degree of membrane oxidative damage and regulate ferroptosis sensitivity. PUFA substrates, including epinephrine (AdA) and arachidonic acid (AA), are indispensable substances for peroxidation to occur during ferroptosis (Kagan et al., 2017). And the key enzymes involved in lipid oxidation include long-chain acyl-CoA synthetase member 4 (ACSL4), lysophosphatidylcholine acyltransferase 3 (LPCAT3), and lipoxygenases (LOXs). Initially, ACSL4 converts AA and AdA into acyl-CoA derivatives, which are subsequently incorporated into phosphatidylethanolamine (PUFA-PE) by LPCAT3 (Ursini and Maiorino, 2020). LOXs then oxidize PUFA-PE to generate lipid hydroperoxides (PLOOH) (Jiang et al., 2021). If not reduced to corresponding alcohols (PLOH) by GPX4, PLOOH species continuously produce free radicals through Fenton reaction. During this process, the accumulation of PLOOH triggers radical chain reactions that compromise PUFA-rich membranes, promoting membrane and organelle rupture, and accelerating ferroptosis (Yang W. S. et al., 2016). High levels of lipid hydroperoxides promote ferroptosis, and these high levels are inseparable from the upregulation of ACSL4, LPCAT3 or LOXs expression levels (Zou et al., 2019). Thus, inhibiting these enzymes may reduce lipid peroxidation and delay the onset of ferroptosis. Doll et al. demonstrated that knockdown of ACSL4 or LPCAT3 inhibits ferroptosis, with ACSL4 suppression exerting a more pronounced effect (Doll et al., 2019) (Figure 2).

### 2.3 Amino acid metabolism

System Xc−, a cystine/glutamate antiporter comprising the catalytic subunit SLC7A11 and the regulatory subunit SLC3A2, is broadly expressed on the plasma membrane (Koppula et al., 2021; Fang et al., 2023). It imports extracellular cystine into the cell in exchange for intracellular glutamate at a 1:1 ratio (Jiang et al., 2021). Cysteine, together with glutamate and glycine, serves as a substrate for enzymatic GSH synthesis (Bersuker et al., 2019). Additionally, previous studies have shown that GPX4 is a GSH-dependent enzyme that reduces PLOOH to PLOH, thereby preventing lipid peroxidation and inhibiting ferroptosis. Inhibition of GPX4 catalytic activity disrupts redox homeostasis and induces AKI in mice, as demonstrated by Friedmann Angeli et al. (2014).

As typical inducers of ferroptosis, Erastin and RSL3 trigger cellular oxidative stress through distinct molecular mechanisms. Erastin specifically inhibits System Xc−, depleting GSH, whereas RSL3 targets the active site of GPX4, disrupting antioxidant defenses and promoting the pathological accumulation of ROS (Floros et al., 2021). Collectively, these components constitute the System Xc−–GSH–GPX4 metabolic axis, a central pathway in ferroptosis regulation. Damage to System Xc− or disruption of GSH synthesis can reduce GPX4 activity, disturb redox homeostasis and trigger ferroptosis (Figure 2).

### 2.4 Nrf2 pathway (p62-Keap1-Nrf2 signaling pathway)

Nuclear factor erythroid 2–related factor 2 (Nrf2) is a transcription factor that activates genes with antioxidant response elements (AREs), thereby maintaining cellular redox balance and protecting against iron-dependent cell death. Under normal physiological conditions, Nrf2 binds to Keap1 in the cytoplasm, which facilitates its ubiquitination and subsequent degradation, thus suppressing Nrf2 activity (Cuadrado et al., 2019). However, oxidative stress or imbalances in iron metabolism cause conformational changes in Keap1, allowing Nrf2 to escape degradation. Nrf2 then translocates to the nucleus, where it interacts with AREs and activates the transcription of genes involved in antioxidant defense to counteract oxidative stress (He et al., 2020). Additionally, phosphorylated p62/SQSTM1 binds to Keap1 with high affinity, promoting Nrf2 release from the Keap1 complex (Sun et al., 2016). Thus, the p62-Keap1-Nrf2 signaling axis positively regulates Nrf2 activity, enhancing cellular antioxidant defenses.

Studies indicate that Nrf2 inhibits ferroptosis by regulating iron homeostasis, glutathione metabolism, and intermediary metabolism. Nrf2 activates antioxidant genes like HO-1 and FTH1, maintaining iron homeostasis and reducing ferroptosis sensitivity (Dong et al., 2020; Anandhan et al., 2023). Additionally, Nrf2 regulates ferroptosis-related proteins and enzymes, including GPX4, SLC7A11, and glutathione synthetase, further inhibiting ferroptosis (Abdalkader et al., 2018). Another study reported that Nrf2 directly enhances the transcription of G6PD and IDH1, coordinating the increase of NADPH production. This process supports essential metabolic processes, including antioxidant defense, reductive biosynthesis (e.g., fatty acid synthesis), and maintenance of redox homeostasis (Yang H.-C. et al., 2016). Research indicates that activating Nrf2 in renal tubular epithelial cells can alleviate the pathological progression of AKI. Targeted intervention in the Keap1-Nrf2 axis significantly suppresses oxidative damage cascades in renal tissue, thereby hindering the progression of chronic kidney disease (Nezu et al., 2017) (Figure 2).

### 2.5 Auxiliary mechanisms of ferroptosis in AKI

Beyond the four primary pathways, ferroptosis in AKI is also regulated by additional auxiliary factors. These regulatory factors include p53, heat shock proteins (HSPs), and Hippo-YAP/TAZ pathway. It is well established that ferroptosis in AKI arises from disrupted antioxidant defenses and enhanced lipid peroxidation. A recent study shows that p53-mediated suppression of SLC7A11 intensifies oxidative stress, while HSPB1 and HSPA5 preserve GPX4 activity and cytoskeletal integrity to reduce cellular injury. Furthermore, the Hippo-YAP/TAZ pathway regulates antioxidant gene expression and ferroptotic sensitivity in AKI via cell-density signal (Han et al., 2024).

### 2.6 Cell-type specificity of ferroptosis in AKI

Recent studies indicate that different renal cell types vary significantly in their sensitivity to ferroptosis, suggesting a high degree of cell-type-specific regulation. This specificity plays a critical role in understanding the pathogenesis of AKI and developing effective treatment strategies.

Proximal tubular epithelial cells (PTECs), which are highly susceptible in AKI, show pronounced sensitivity to ferroptosis. PTECs exhibit robust iron uptake and reabsorption capabilities, leading to elevated intracellular iron levels. This iron overload facilitates Fenton reactions, producing excessive ROS and initiating lipid peroxidation (Wang et al., 2025). Moreover, PTECs have membranes rich in PUFAs and limited antioxidant defenses under stress, rendering them especially prone to ferroptosis in ischemia-reperfusion injury (IRI) and cisplatin-induced AKI models. Evidence indicates that following IRI, key anti-ferroptotic proteins like GPX4 and SLC7A11 are downregulated in PTECs, whereas the pro-ferroptotic factor ACSL4 is upregulated, intensifying lipid peroxidation and cellular damage (Poindessous et al., 2024; Zuo et al., 2024).

In contrast, distal tubular cells, podocytes, endothelial cells, and interstitial fibroblasts exhibit greater resistance to ferroptosis. This resistance is primarily attributed to their efficient antioxidant systems and stringent regulation of iron homeostasis. In these cells, Nrf2 is more active, promoting the expression of antioxidant genes like SLC7A11 and GPX4. It also upregulates iron-handling genes, facilitating the clearance of excess iron and ROS (Yan et al., 2023). Additionally, these cells contain membrane lipids with higher levels of saturated and monounsaturated fatty acids (MUFAs), conferring resistance to lipid peroxidation. Experimental evidence demonstrates that MUFA supplementation, such as oleic acid, decreases membrane PUFA content and significantly inhibits ferroptosis (Magtanong et al., 2019).

The varying sensitivity of renal cell types to ferroptosis provides new therapeutic insights for AKI. Specifically, targeted strategies may protect susceptible PTECs. Multiple studies suggest that ferroptosis inhibitors and iron chelators are promising in alleviating kidney injury (Sun et al., 2023). Additionally, dietary or pharmacological MUFA supplementation may bolster the ferroptosis resistance of other tubular cell types (Magtanong et al., 2019). Furthermore, integrating various interventions based on the ferroptosis susceptibility of specific renal regions could facilitate more precise treatments. Current research explores utilizing ferroptosis-related genes or biomarkers to guide personalized therapeutic strategies.

To sum up, inherent differences in iron metabolism, lipid composition, and antioxidant capacity among renal cell types significantly influence their sensitivity to ferroptosis (Wang et al., 2025). Future studies should delve deeper into these mechanisms to develop more precise and personalized interventions for AKI.

## 3 Ferroptosis and various types of AKI

**FIGURE 3:**
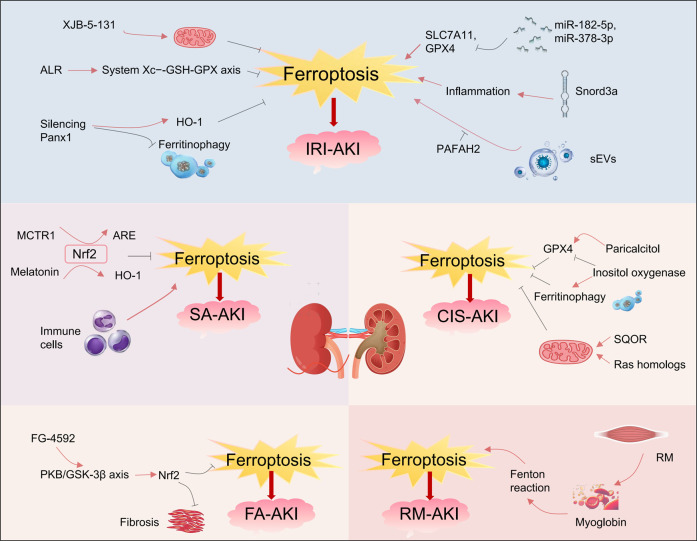
Ferroptosis and various types of acute kidney injury. Ferroptosis occurs in various types of acute kidney injury, including ischemia‒reperfusion injury induced AKI (IRI-AKI), sepsis-induced AKI (SA-AKI), folic acid induced AKI (FA-AKI), cisplatin-induced AKI (CIS-AKI) and rhabdomyolysis-induced AKI (RM-AKI). Within these types of AKI, numerous small molecules can either inhibit or promote ferroptosis, thereby influencing the progression of AKI. In this picture, the red arrows represent promotion and the black ones represent inhibition. ALR, Augmenter of liver regeneration; Panx1, Pannexin1; Snord3a, The small nucleolar RNA family member; sEV, Secretory extracellular vesicles; MCTR1, Macrophage-derived mediator; SQOR, Sulfide-quinone oxidoreductase; FG-4592, The hypoxia-inducible factor stabilizer.

AKI refers to a sudden loss of kidney function caused by diverse causes. Increasing evidence indicates that ferroptosis plays a critical role in the pathogenesis of AKI. This section reviews recent advances in understanding the role of ferroptosis in different types of AKI (Figure 3).

### 3.1 Ferroptosis and ischemia-reperfusion injury-induced AKI

Ischemia-reperfusion injury (IRI) is a key pathophysiological mechanism in AKI, with ferroptosis playing a crucial role in IRI-related AKI (IRI-AKI). Linkermann et al. showed that the third-generation ferroptosis inhibitor Fer-16-86 effectively interrupts the ferroptosis pathway in renal tubular epithelial cells. In IRI-AKI models, Fer-16-86 improved renal function markers, including serum creatinine and urea nitrogen, and reduced kidney tissue injury scores (Linkermann et al., 2014). Additionally, another ferroptosis inhibitor, XJB-5-131, exerts dual effects of mitochondrial targeting and free radical scavenging. By neutralizing lipid peroxides and stabilizing mitochondrial membrane potential, XJB-5-131 precisely regulates the ferroptosis pathway, alleviating IRI-related kidney damage (Zhao et al., 2020). Recent studies have identified the augmenter of liver regeneration (ALR) as a specific antagonist of ferroptosis. In IRI mouse models, ALR reduced ferroptosis incidence in renal tubules by scavenging free radicals and modulating the System Xc−-GSH-GPX antioxidant axis (Huang et al., 2019; Ni et al., 2022).

At the level of epigenetic regulation, microRNAs (miRNAs) and snoRNAs also play crucial roles in modulating cellular ferroptosis. In IRI-induced kidney damage, upregulated miR-182-5p and miR-378-3p suppress the expression of SLC7A11 and GPX4 proteins. This suppression creates a molecular environment that promotes ferroptosis, thereby accelerating the pathological progression of IRI-AKI (Ding et al., 2020). As a small nucleolar RNA family member, Snord3a reorganizes the iron metabolism-inflammation interaction system. In both IRI-AKI and cisplatin-induced AKI models, Snord3a drives ferroptosis in renal tubular epithelial cells by aberrantly activating oxidative stress signaling pathways (Zhu et al., 2024).

A recent study demonstrated that pannexin 1 (Panx1) is a channel protein widely expressed in tissues, including the kidney. Silencing Panx1 increases HO-1 expression and alleviates ferritinophagy, thereby protecting mice from IRI-AKI damage (Su et al., 2019). Moreover, in hypoxic kidney injury, human proximal tubular epithelial cells release pathological secretory extracellular vesicles (sEVs) that contribute significantly to the progression of kidney injury. These vesicles trigger lipid peroxidation cascades that promote ferroptosis. They also promote ferroptosis in adjacent tubular cells, accelerating the progression from AKI to CKD. Notably, platelet-activating factor acetylhydrolase (II) (PAFAH2) specifically blocks the ferroptosis pathway involved in this process, mitigating kidney damage from IRI (Wang et al., 2024; Zhang Q. et al., 2024).

### 3.2 Ferroptosis and sepsis-induced AKI

Sepsis is characterized by an imbalance in immune homeostasis, which leads to multi-organ dysfunction. AKI is one of the most clinically challenging complications of sepsis, often progressing into sepsis-specific acute kidney injury (SA-AKI). Research suggests that the mechanism of iron-dependent cell death plays a central regulatory role in SA-AKI mouse models. In sepsis mouse models, serum iron levels are elevated, and ferritin synthesis increases. Moreover, regulating ferroptosis-related genes and pathways significantly impacts the progression of kidney injury (Xiao et al., 2021). Nrf2, a key regulator of redox homeostasis, effectively inhibits ferroptosis by maintaining redox balance and modulating iron metabolism. Xiao et al. showed that the macrophage-derived mediator MCTR1 significantly improves kidney function in the SA-AKI model by activating the Nrf2-ARE axis (Xiao et al., 2021). Furthermore, melatonin treatment activates the Nrf2/HO-1 signaling pathway, enhancing the expression of antioxidant genes like GPX4 and SLC7A11. This activation suppresses lipid peroxidation, thereby reducing ferroptosis and alleviating kidney injury in sepsis models (Qiu et al., 2022).

It is noteworthy that the inflammatory responses of immune cells also play an important role in regulating the process of ferroptosis. Excessive neutrophil activation promotes the development of AKI by damaging vascular permeability and endothelial function (Akcay et al., 2009). Moreover, the level of regulatory T cell (Treg) infiltration is negatively correlated with the expression of multiple ferroptosis-promoting genes (Guo et al., 2022). In sepsis-related AKI, neutrophil infiltration increases, whereas Treg infiltration decreases. These changes drive the pathological progression of SA-AKI through key molecular events underlying ferroptosis (Guo et al., 2022). Conversely, macrophages enhance kidney function in SA-AKI mice via the ARE regulatory axis (Xiao et al., 2021). In summary, understanding the regulatory mechanisms of ferroptosis and its interactions with the immune microenvironment offers valuable insights and promising therapeutic targets for developing novel treatments for SA-AKI, including Nrf2 pathway agonists, immune cell modulators, and iron chelators.

### 3.3 Ferroptosis and cisplatin-induced AKI

Cisplatin is a widely utilized broad-spectrum chemotherapeutic agent. Nevertheless, its clinical utility is significantly constrained by dose-dependent nephrotoxicity, particularly under conditions of prolonged high-dose administration, which leads to renal cellular injury. Studies indicate that ferroptosis is a critical molecular event in cisplatin-induced nephrotoxicity. Sharma et al. showed that ferroptosis inhibitors, including deferoxamine and iron chelator-1, improve both renal function and histological integrity in cisplatin-induced AKI, confirming the central role of ferroptosis in this disease (Ikeda et al., 2021). GPX4 serves as a key regulatory factor in the ferroptosis process, primarily responsible for detoxifying lipid peroxides. It has been reported that its agonist, paricalcitol, upregulates GPX4 expression levels to restore antioxidant defense capacity, significantly reducing ferroptosis in cisplatin-induced kidney damage (Hu et al., 2020). Moreover, inositol oxygenase, expressed in proximal tubules, aggravates ferroptosis in cisplatin-induced AKI by suppressing GPX4, increasing lipid peroxidation, and promoting ferritinophagy (Dutta et al., 2017).

The induction of ferroptosis by cisplatin is also modulated by mitochondrial structural integrity and metabolic activity. As key intracellular iron reservoirs, mitochondria can promote ferroptotic signaling by releasing excess iron upon functional disruption. Sulfide-quinone oxidoreductase (SQOR), a mitochondrial membrane protein complex, is specifically expressed in the kidney and plays a role in mitochondrial energy metabolism. Recent studies have shown that SQOR knockout disrupts mitochondrial energy metabolism, significantly exacerbating the ferroptosis process in renal tubules induced by cisplatin. Moreover, targeting SQOR may represent a novel therapeutic strategy for nephrotoxic AKI (Cai et al., 2024). At the molecular regulatory level, it has been shown that Ras homologs (Rheb1) enriched in the brain inhibit renal ferroptosis by preserving mitochondrial membrane integrity and enhancing oxidative phosphorylation, thereby alleviating cisplatin-induced kidney cell injury (Lu et al., 2020).

### 3.4 Ferroptosis and folic acid-induced AKI

Folic acid (FA) is another common nephrotoxic drug, inducing AKI in a dose-dependent biphasic manner. Hwang et al. reported that physiological doses of FA alleviate oxidative stress, while supraphysiological levels damage renal tubular epithelial cells (Hwang et al., 2011; Ducker and Rabinowitz, 2017). In folic acid-induced AKI mouse models, Martin-Sanchez et al. observed substantial lipid peroxidation in renal tissue. Treatment with the ferroptosis inhibitor Fer-1 preserved renal function and reduced tissue injury, supporting ferroptosis as a major contributing mechanism in FA-induced AKI (Martin-Sanchez et al., 2017). Stable HIF-1α, as an endogenous regulator of oxidative stress, activates the downstream Nrf2 pathway, thereby regulating the expression of antioxidant enzymes and iron metabolism proteins. FG-4592 (Roxadustat) is a PHD (prolyl hydroxylase) inhibitor that stabilizes HIF-1α by inhibiting PHD activity, preventing the hydroxylation and degradation of the HIF-α subunit under normoxic conditions (Li et al., 2020). Further research revealed that the hypoxia-inducible factor stabilizer FG-4592 modulates ferroptosis by regulating iron metabolism, lipid peroxidation, and antioxidant defenses. In the FA-AKI model, FG-4592 reduced renal iron levels and downregulated ferroptosis markers including MDA and 4-HNE, alleviating FA-induced kidney inflammation and renal cell ferroptosis. This study also demonstrated that activated protein kinase B (PKB/AKT) phosphorylates and inhibits glycogen synthase kinase 3β (GSK-3β). The inactivation of GSK-3β relieves its negative regulation on the transcription factor Nrf2, promoting Nrf2 stability and its translocation to the nucleus. Subsequently, activated Nrf2 significantly enhances the expression of downstream key antioxidant proteins, such as GPX4 and HO-1. This signaling cascade effectively suppresses the fatty acid-induced ferroptosis process and delays the progression of kidney fibrosis (Li et al., 2020). In conclusion, these studies consistently confirm that the ferroptosis signaling pathway is clearly activated in FA-AKI and plays a central role in disease progression.

### 3.5 Ferroptosis and rhabdomyolysis-induced AKI

Rhabdomyolysis (RM) is a severe systemic condition that frequently leads to the development of AKI. The breakdown of striated muscle cells releases large quantities of myoglobin into circulation, where it accumulates in the kidneys. The degradation of myoglobin generates Fe^2+^, which triggers a cascade of lipid peroxidation via the Fenton reaction, leading to ferroptosis in renal tissue and driving the development of AKI (Zhang J. et al., 2021). Research by Guerrero-Hue et al. demonstrated that ferroptosis inhibitors (e.g., Fer-1) or iron chelators (e.g., deferoxamine) reduce kidney inflammation and oxidative stress markers in RM-AKI models, while improving renal function, as indicated by serum creatinine clearance (Guerrero-Hue et al., 2019). Additionally, Zarjou et al. established a FTH1 knockout model and found that FTH1 deficiency exacerbated RM-induced renal injury. These findings highlight the therapeutic potential of targeting ferroptosis-associated pathways to mitigate renal injury in AKI (Zarjou et al., 2013). These findings indicate that ferroptosis plays a central role in the pathogenesis and progression of AKI induced by RM. Targeting these pathways may provide therapeutic benefits.

## 4 Therapeutic strategies targeting ferroptosis in AKI

Ferroptosis plays a pivotal role in the pathogenesis of AKI. Clinical evidence shows that excessive urinary accumulation of iron-dependent phospholipid hydroperoxides (Fe-PUFA-OOH) in AKI patients is inversely associated with renal functional recovery. These findings suggest that modulating the ferroptosis signaling pathway could offer a promising therapeutic strategy for AKI (Liang et al., 2023). Current therapeutic approaches aim to regulate ferroptosis through multiple mechanisms, including radical-trapping antioxidants, iron chelators, ACSL4 inhibitors, and lipophilic antioxidants.

### 4.1 Radical-trapping antioxidants

Ferrostatin-1 (Fer-1), the first-generation synthetic ferroptosis inhibitor, effectively suppresses ferroptosis by scavenging ROS and inhibiting lipid peroxidation (Martin-Sanchez et al., 2017). However, its efficacy and stability *in vivo* are relatively low, which significantly limits its clinical applicability. Linkermann et al. identified a third-generation small-molecule ferroptosis inhibitor, 16-86, which demonstrates improved pharmacokinetics compared to Fer-1. In a murine model of IR-AKI, 16-86 significantly reduces serum creatinine levels and attenuates tubular interstitial damage (Doll et al., 2019).

Liproxstatin-1 (Lip-1) is another potent antioxidant that effectively inhibits ferroptosis by scavenging free radicals and preventing iron-mediated lipid peroxidation. Compared to Fer-1, Lip-1 shows enhanced stability *in vivo*. At low doses, Lip-1 selectively disrupts ferroptosis by inhibiting the formation of lipid hydroperoxides, without affecting other forms of cell death such as apoptosis or pyroptosis (Zhang B. et al., 2021). In ischemia-reperfusion injury models, Friedmann Angeli et al. demonstrated that Lip-1 significantly suppresses ferroptosis-associated markers in renal tubular epithelial cells (Friedmann Angeli et al., 2014). Subsequent investigations revealed that in a rat model of AKI, Lip-1 treatment reduced lipid peroxidation byproducts and lowered tubular interstitial injury scores in renal tissues, underscoring its therapeutic potential against iron-dependent oxidative injury (Ma et al., 2021).

### 4.2 Iron chelators

Research indicates that iron chelators, including deferiprone, deferoxamine (DFO), and ciclopirox, can alleviate rhabdomyolysis-induced AKI. The underlying mechanisms involve the inhibition of ferroptosis by reducing free iron and limiting Fenton reaction-driven lipid peroxidation (Cao and Dixon, 2016). However, their clinical application is limited by a range of adverse effects. Deferiprone (DFP), an oral alternative to DFO, has been used in clinical settings. However, its precise renoprotective effects remain unclear and require further investigation. In animal models, DFP reduces iron accumulation in the renal cortex by 58% compared to controls, although the improvement in glomerular filtration rate is only 12%. This suggests that DFP’s renoprotective mechanism may be tissue-specific (Groebler et al., 2011; Zhang et al., 2022).

### 4.3 ACSL4 inhibitors

Preclinical studies have demonstrated that thiazolidinedione drugs—such as rosiglitazone, pioglitazone, and ciglitazone—inhibit ACSL4 activity, thereby disrupting the synthesis of phospholipids that promote ferroptosis (e.g., PE-AA/AdA). This intervention prevents ferroptosis in GPX4-deficient animal models and significantly enhances survival rates in treated groups (Kagan et al., 2017). Recent pharmacological studies have identified AS-252424 as a selective inhibitor of ACSL4. This compound confers significant protection against organ damage in models of acute kidney and liver injury by disrupting the ACSL4-mediated lipid peroxidation signaling pathway (Huang et al., 2024).

### 4.4 Lipophilic antioxidants

Mishima et al. demonstrated that vitamin D receptor agonists provide significant renal protection in a cisplatin-induced AKI model by selectively eliminating lipid peroxidation byproducts (Mishima et al., 2020). Vitamin E, a lipophilic antioxidant, mitigates ferroptosis by maintaining the renal antioxidant environment and inhibiting free radical chain reactions, thus alleviating AKI caused by ischemia-reperfusion (Shimizu et al., 2004). Recent nanotechnology studies indicate that ROS/MMP2 dual-responsive nanocarriers can target damaged renal cells and deliver vitamin E, significantly reducing ROS and lipid peroxidation products (MDA, 4-HNE) (Zhang J. et al., 2024). Furthermore, vitamin K1 regulates ferroptosis by upregulating GPX4 activity and promoting the coordinated expression of ferroptosis suppressor protein 1 (FSP1) and dihydroorotate dehydrogenase (DHODH), thus inhibiting ferroptosis induced by RSL3 or Erastin. Vitamin K1 may also protect against AKI induced by IRI by reprogramming iron metabolism (Kolbrink et al., 2022).

## 5 Discussion and perspectives

In conclusion, ferroptosis is a recently recognized form of iron-dependent cell death, characterized by intracellular iron accumulation and lipid peroxidation. This review outlines the molecular mechanisms of ferroptosis, its involvement in various forms of AKI, and related therapeutic strategies. However, the role of ferroptosis in AKI pathogenesis remains poorly understood. Advances in gene-editing and targeted therapies may lead to novel therapeutic breakthroughs. Recent studies highlight the potential of gene-editing tools to regulate ferroptosis-associated genes. By using the CRISPR-dCas13d-eIF4G platform, researchers enhanced GPX4 expression in renal epithelial cells, which reduced calcium oxalate-induced ferroptosis and kidney stone formation in both human cells and mouse models (He et al., 2024). Future efforts should focus on refining CRISPR-based therapies and advancing gene-editing strategies to precisely regulate ferroptosis genes in renal tissue.

Recently, several small-molecule compounds have been identified as potential therapeutic agents for AKI in preclinical models. However, their safety and efficacy require validation in future preclinical and clinical trials, and pathways for clinical translation remain to be defined (Jun et al., 2020). Furthermore, the specific biomarkers for ferroptosis remain unidentified, in contrast to apoptosis (caspase activation) and autophagy (autolysosome formation). Early studies mainly examined non-specific biomarkers of ferroptosis, including NRF2, GPX4, FTH, NCOA4, and HO-1 (Ni et al., 2022). Recent research indicates that advances in multi-omics and spatial profiling show promise in identifying ferroptosis-specific biomarkers, which will require further investigation in the coming years (Bayır et al., 2023; Lake et al., 2023). In conclusion, as a central mechanism in AKI pathogenesis, ferroptosis represents a promising novel therapeutic target.
